# Corrigendum: Number of Childbearing Partners, Status, and the Fertility of Men and Women in the U.S.

**DOI:** 10.3389/fsoc.2019.00090

**Published:** 2020-02-06

**Authors:** Rosemary L. Hopcroft

**Affiliations:** Sociology, University of North Carolina, Charlotte, NC, United States

**Keywords:** sex differences, fertility, evolutionary theory, SIPP data, status

In the original article, there was a mistake in Figure 1 and Figure 2 as published. A copying error was made in Excel, however, the numbers in Table 5, the source for the figures, are correct. The corrected Figure 1 and Figure 2 appear below.

**Figure 1:**
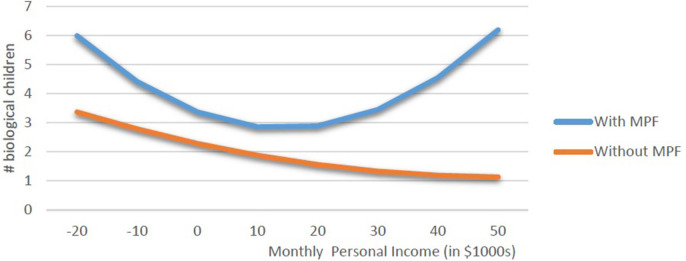
Biological children, women with and without multipartner fertility.

**Figure 2:**
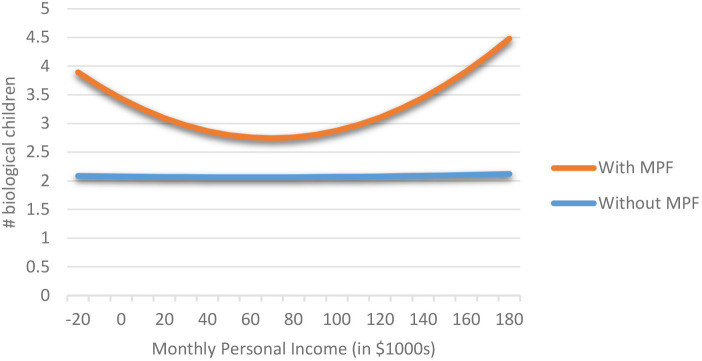
Biological children, men with and without multipartner fertility.

The author apologizes for this error and states that this does not change the scientific conclusions of the article in any way. The original article has been updated.

